# Multiple Undifferentiated Pleomorphic Sarcoma (Malignant Fibrous Histiocytoma) with Extradural Involvement in a 7-Year-Old Labrador Retriever

**DOI:** 10.3390/vetsci9010003

**Published:** 2021-12-23

**Authors:** Kihoon Kim, Jaiho Shin, Hyosung Kim, Hanjun Kim, Jaehwan Kim, Sunhee Do, Hwiyool Kim

**Affiliations:** 1 Department of Surgery, College of Veterinary Medicine, Konkuk University, Seoul 05029, Korea; kilokpi@konkuk.ac.kr; 2 Cheongna Gongam Animal Hospital, Incheon 22737, Korea; kuvet@naver.com; 3 Department of Clinical Pathology, College of Veterinary Medicine, Konkuk University, Seoul 05029, Korea; w6373515@naver.com (H.K.); shdo@konkuk.ac.kr (S.D.); 4 Terasaki Institute for Biomedical Innovation, Los Angeles, CA 90064, USA; hkim@terasaki.org; 5 Department of Veterinary Medical Imaging, College of Veterinary Medicine, Konkuk University, Seoul 05029, Korea; jaehwan@konkuk.ac.kr

**Keywords:** dog, extradural, spinal cord, undifferentiated pleomorphic sarcoma

## Abstract

A 7-year-old castrated male Labrador retriever was referred for evaluation of progressive hind limb paresis of 4 weeks’ duration. On computed tomography and magnetic resonance imaging examination, masses were found in several regions including the lung, right kidney, and peritoneum. Additionally, an extradural mass at the region of T13–L1 was identified, which is assumed to related to the chief complaint, progressive hind limb paresis. With the consent of the owner, a dorsal laminectomy was performed to remove the mass and surrounding tissues for the palliation of the hind limb paresis. Hematoxylin and eosin staining and immunohistochemical examination revealed the mass to be consistent with an undifferentiated (high-grade) pleomorphic sarcoma. The patient presented with recurrence of the hind limb paresis, respiratory discomfort, and urinary incontinence. The owner declined treatment and the dog was euthanized. Systemic metastasis was confirmed on postmortem microscopic examination. To the authors’ knowledge, this is the first report describing multiple undifferentiated high-grade pleomorphic sarcoma with extradural involvement developing into the vertebral canal through the intervertebral space, resulting in spinal damage, in a dog.

## 1. Introduction

Undifferentiated pleomorphic sarcomas (UPSs), previously known as “malignant fibrous histiocytomas” (MFHs) are a group of soft tissue sarcomas reported in several species including humans, dogs, and cats [1,2,3,4]. Although no breed predisposition is known, flat coated retrievers, rottweilers, and golden retrievers with UPSs are frequently reported previously [2].

According to the human literature, UPSs are known as an expansile tumor, usually found at the retroperitoneum or extremities. They may infiltrate to surrounding tissues. Metastasis to lung, liver, lymph node, and bone has been also reported [5]. In veterinary literature, UPSs are known to occur in the soft tissues of the limbs or trunk, spleen, liver, kidney, mesenteric lymph nodes, and skull in middle-aged to older dogs [2,6,7]. 

Although extradural UPSs have rarely been reported in the human literature, to the best of our knowledge, they have not been reported in veterinary medicine to date. This case report describes multiple UPS with extradural involvement and the surgical management of an extradural UPS, which was differentiated from other malignant sarcomas using immunohistochemical (IHC) staining, affecting the spinal cord in a dog. Furthermore, the current study reports on the systematic metastasis identified on postmortem microscopic examination. 

## 2. Clinical Description

A 7-year-old castrated male Labrador retriever dog presented with progressive ataxia and hind limb paresis of 1 month duration. On physical examination, the dog was bright, alert, and responsive but had an inability to climb stairs, bilateral hindlimb lameness, and mild bilaterally symmetric muscle atrophy of the gluteal, quadriceps, and caudal thigh muscles. Neurologic examination indicated that the forelimbs were normal, but postural reactions of the hindlimbs, including the proprioception position, hopping, hemistanding, hemiwalking, and extensor postural thrust, were decreased (Table 1). Spinal reflexes of the hind limb, including the cranial tibial and withdrawal reflexes, were also diminished (Table 2). Hematological and serum biochemical analysis were unremarkable. 

**Figure 1:**
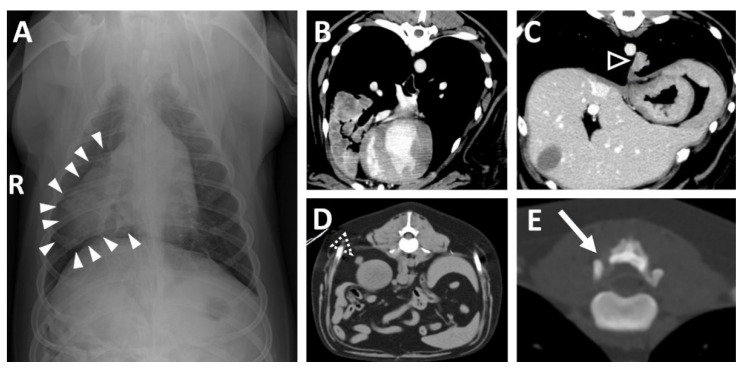
(**A** Soft tissue opacity (arrowheads) in the right middle lung lobe on thoracic radiography. (**B**) Large mass of the right middle lung lobe and (**C**) small mass of the left caudal lung lobe (open arrowhead) on post-contrast transverse images. (**D**) Small mass (open dashed arrowhead) dorsal to the right kidney. (**E**) Osteolytic lesion (arrow) of the T13. (R: right, T13: the 13rd thoracic vertebra).

Thoracic radiography revealed a small, round soft tissue opacity in the right middle lung lobe (Figure 1A) and a lytic bone lesion adjacent to the articular facets between the thirteenth thoracic vertebra (T13) and first lumbar vertebra (L1). Abdominal ultrasonographic examination was unremarkable. Computed tomography (CT, Lightspeed, GE Healthcare, Milwaukee, WI, USA) was performed to evaluate the vertebral lesion and the mass in the lung, and CT images were obtained before and after intravenous administration of a contrast medium (Omnipaque 300; GE Healthcare, Milwaukee, WI, USA). A post-contrast CT scan revealed small soft tissue masses in the right middle lung lobe (Figure 1B) and left caudal lobe (Figure 1C). A small soft tissue mass caudal to the right kidney was also found (Figure 1D). Moreover, osteolytic changes of the vertebral lamina and pedicle of T13 were identified (Figure 1E). 

**Figure 2:**
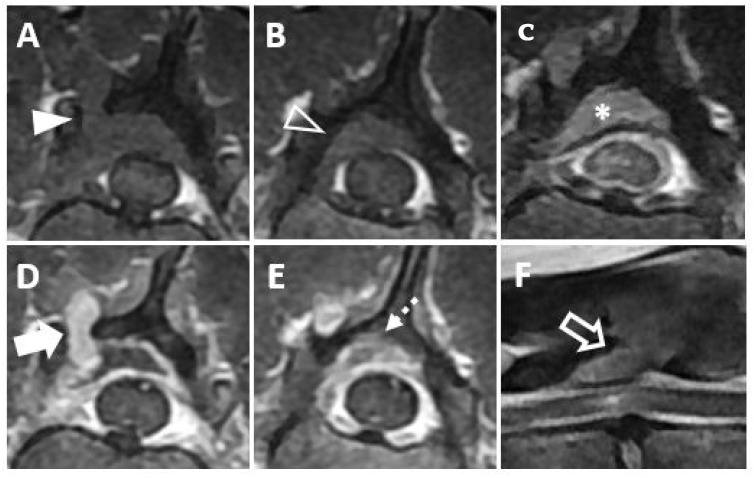
Magnetic resonance images showing a mass invading the vertebral canal and the spinal cord lesion are depicted. (**A** The mass appears iso-intense at the bone lysis region on a transverse T1-weighted image (arrowhead). (**B**) The mass can be seen invading the vertebral canal on a transverse T1-weighted image (open arrowhead). (**C**) The mass can be seen mildly compressing the spinal cord (asterisk) and contrast enhancement of the spinal cord parenchyma (dashed arrowhead) is visible on a post-contrast, transverse T1-weighted image. (**D**) Contrast enhancement of the mass (arrow) is shown on a post-contrast, transverse T1-weighted image. (**E**) The mass can be seen invading the vertebral canal on a post-contrast, transverse T1-weighted image (dashed arrow). (**F**) The mass (open arrow) between T13 and L1 causing hyperintensity of the spinal cord parenchyma is shown on a sagittal T2-weighted image. (T13: the thirteenth thoracic vertebra, L1: the first lumbar vertebra.).

Magnetic resonance imaging (MRI, 1.5T, Signa, GE Healthcare, Waukesha, WI, USA) was performed before and after intravenous administration of a contrast medium (Magnevist; Beyer-Schering Pharmaceutical, Berlin, Germany). The mass was identified at the bone lysis region on a transverse T1-weighted image (Figure 2A) and on a post-contrast, transverse T1-weighted image (Figure 2D). The mass was seen to be invading the vertebral canal on the transverse T1-weighted image (Figure 2B) and on the post-contrast, transverse T1-weighted image (Figure 2E). On the post-contrast, transverse T1-weighted image, the mass was found to be mildly compressing the spinal cord and contrast enhancement of the spinal cord parenchyma was identified, suggesting a mild inflammatory response to the mass (Figure 2C). On a sagittal T2-weighted image, hyperintensity of the spinal cord parenchyma was identified (Figure 2F). Based on CT and MRI findings, an extradural mass invading the vertebral canal was diagnosed. Surgical management of the mass was performed, with the informed consent of the owner, for palliation of hind limb paresis, despite the multiple masses involving pulmonary region.

The dog was premedicated with butorphanol (0.5 mg/kg, Butophan; Myungmoon Pharmaceutical, Seoul, Korea) and midazolam (0.1 mg/kg, Midazolam; Bukwang Pharmaceutical, Seoul, Korea) before anesthetic induction with propofol (4 mg/kg, Provive Inj.; Myungmoon Pharmaceutical, Seoul, Korea). After intubation, general anesthesia was maintained with isoflurane (Isoflurane; Choongwae Pharmaceutical, Seoul, Korea) with oxygen. 

With the patient positioned in sternal recumbency, a dorsal midline incision was made under C-arm guidance to accurately expose T13 and L1. A dorsal laminectomy was performed for tumor resection. A rongeur was used for removal of the dorsal lamina, a portion of T13, the L1 spinous process, and the mass surrounding the spinal cord. The remaining dorsal fragment of the vertebral arch was removed with a pneumatic high-speed drill (i-Medicom, Seoul, Korea) and 3 mm round bur (i-Medicom, Seoul, Korea) for exposure of the spinal cord. The removal of the mass with surrounding tissue was continued until the spinal cord was exposed. Skin and soft tissue were closed in a routine manner. Recovery from anesthesia was uneventful. A constant rate infusion of butorphanol (0.2 mg/kg/h, Butophan; Myungmoon Pharmaceutical, Seoul, Korea) was administered after surgery. Postoperative rehabilitation included passive joint ROM exercises of the hindlimbs (starting day 4 and performed 3 to 4 times per day.).

**Figure 3:**
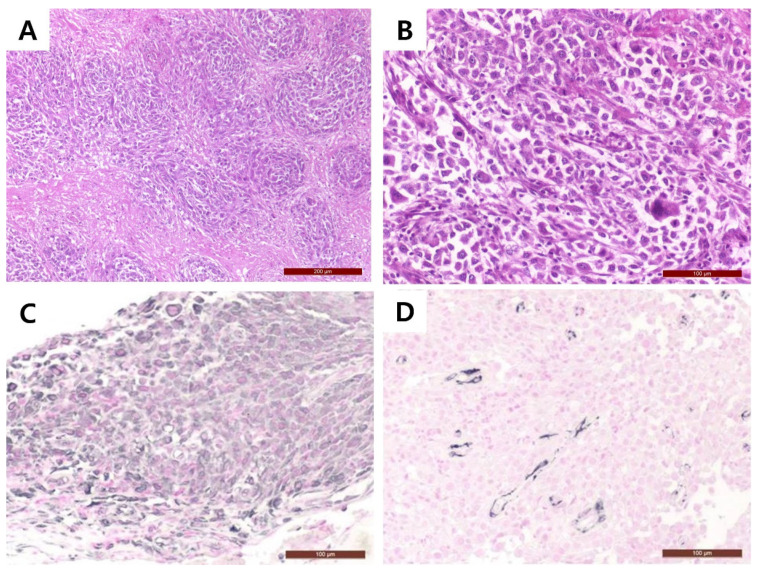
Histopathological examination. (**A**) The arrangement of tumor cells exhibited storiform to fascicular growth pattern. The tumor comprises non-cohesive, disorganized pleomorphic cells with various amounts of eosinophilic cytoplasm. (**B**) Most of the neoplastic cells show moderate to severe nuclear abnormalities, including coarse chromatin patterns, prominent multiple nucleoli, anisokaryosis, and polychromasia. Immunohistochemistry of the mass shows strong (**C**) positive vimentin reactivity and (**D**) negative desmin reactivity. H&E stained tissue sections, (**A**): 40× (**B**): 200×, Immunohistochemistry, (**C,D**) 200×. H&E, hematoxylin and eosin.

The tumor was identified within paravertebral soft tissue and extending into the vertebral canal. The mass was directly attached to the dura mater. The surgically removed tissue samples were fixed in 10% neutral formalin and then routinary processed for histology. From samples paraffin blocks, 4 micron thick were obtained and stained with hematoxylin and eosin (H&E). Microscopic examination revealed the extradural mass invading adjacent tissue structures, causing necrosis. Multifocal areas of mineralization also present within the mass. A haphazard, storiform, and fascicular arrangement of the pleomorphic sarcoma was identified. (Figure 3A). The mass comprised disorganized pleomorphic spindle cells with eosinophilic fibrillar cytoplasm and round-to-oval nuclei that had stippled to clumped chromatin. In addition, the tumor cells showed marked anisocytosis, anisokaryosis, pleomorphism, and bi- or multi-nucleation. Mitoses averaged four per high-power field (Figure 3B). 

Immunohistochemical staining was performed using vimentin (mouse monoclonal, V9, DAKO, Santa Clara, CA, USA, 1:100) as a mesenchymal cell marker, desmin (mouse monoclonal, D33, DAKO, Santa Clara, CA, USA, 1:100) as a smooth and striated muscle cell marker, alkaline phosphatase (rabbit polyclonal, Genetex, Irvine, CA, USA, 1:100) as an osteosarcoma marker, cytokeratin 7 (mouse monoclonal, RCK105, Abcam, Cambridge, UK, 1:1000) as an epithelial marker, S-100 (rabbit polyclonal, DAKO, Santa Clara, CA, USA) as a perineural cell marker, and nestin (mouse monoclonal, Rat401, Abcam, Cambridge, UK, 1:100) as a neural cell marker. Sectioned slides were hydrated through gradient alcohols. Heat-mediated antigen retrieval was performed using microwave in citrate buffer (pH 6.0). Endogenous peroxidase was blocked using 3% H_2_O_2_. Each marker was visualized using vectastain ABC kit (Vector laboratories, Bulrlingame, CA, USA). The signal was visualized with vector SG (Vector laboratories, Bulrlingame, CA, USA) and counterstained with nuclear fast red solution (Vector laboratories, Bulrlingame, CA, USA). Tumor cells showed strong positive staining for vimentin (Figure 3C), while desmin expression was limited to small vessels (Figure 3D). Based on these findings, a diagnosis of UPS was made.

**Figure 4:**
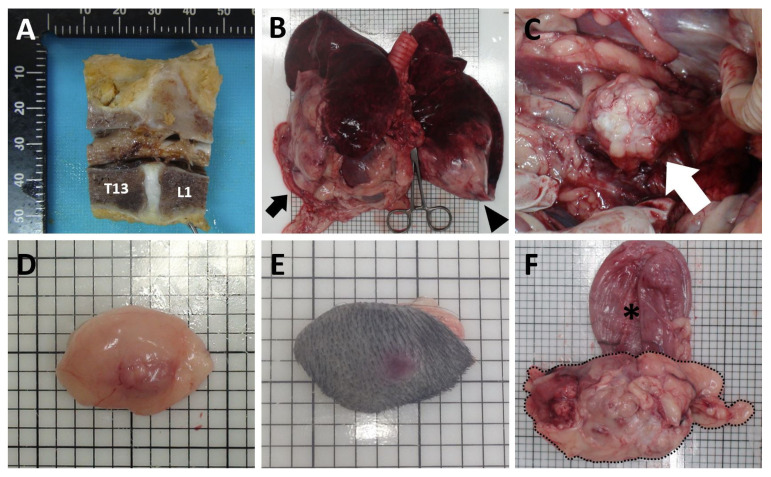
At necropsy, (**A**) recurrence of the mass was found between the T13 and L1. Additionally, the masses were found in the (**B**) right middle and caudal lobe (black arrow) and left caudal lobe (black arrowhead), (**C**) dorsal to the right kidney (white arrow), (**D**) falciform ligament, (**E**) skin, and (**F**) cranial (black dashed line) to the urinary bladder (asterisk).

Proprioception of the left hindlimb returned to normal on the third day after surgery. Gait improvement was identified 4 days after surgery. Chemotherapy was strongly recommended. However, the owner declined additional treatment. The dog was discharged 4 days after surgery and stitches were removed 14 days after surgery. By 6 weeks postoperatively, proprioception of the right hindlimb was improved. Three months after surgery, hind limb paresis recurred, and the patient had difficulty in in breathing and urinary incontinence. Thoracic radiography revealed an enlarged nodule at the right middle lung lobe compared to initial presentation. The dog was euthanized and a complete postmortem examination was performed with the consent of the owner. Recurrence of the mass was identified between the T13 and L1 (Figure 4A). Among the multiple masses at every lung lobe, the most extensive masses were found in right middle and caudal lobe (Figure 4B). Furthermore, masses in the falciform ligament and dorsal to the right kidney were identified (Figure 4C,D). The mass was also identified at the left hind limb (Figure 4E). Moreover, a mass was found cranial to the urinary bladder, which is assumed to be the reason for urinary incontinence (Figure 4F).

**Figure 5:**
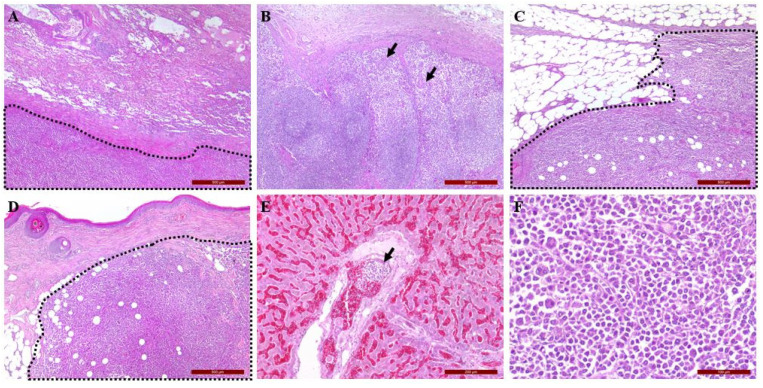
Systemic metastasis of malignant fibrous histiocytoma is illustrated. The tumor cells (black arrow and dash line) can be seen in (**A**,**F**) lung parenchyma; (**B**) hilar lymph nodes of lung; (**C**) peritoneum; (**D**) skin; (**E**) portal vein in the liver. (**A**–**D**) 40×, (**E**) 100×, (**F**) 200×.

Systemic masses including the lung, kidney, hilar lymph nodes, skin, urinary bladder, and peritoneum were confirmed as UPC through H&E and IHC staining, using vimentin antibody. Tumor growth was intensive. The surrounding lung parenchyma was severely inflamed and congested (Figure 5A). In the spleen and lymph nodes, there was little normal tissue due to the infiltration of tumor cells (Figure 5B). Another metastatic neoplasm was found on the peritoneum showing aggressive spread to surrounding tissue (Figure 5C). Distant metastasis was even confirmed on a skin lesion of the left hindlimb (Figure 5D). Interestingly, we could not find obvious metastasis in the liver. However, cellular aggregation with similar cytomorphology was found in the portal vein of the liver, indicating hematogenous dissemination (Figure 5E) Compared to previous findings, the tumor cells found in systemic organs showed a higher nuclear-to-cytoplasm ratio with more frequent multinucleation, indicating more malignant features (Figure 5F).

## 3. Discussion

The term MFH has been used since the early 1960s to describe a group of tumors with a mixed histiocytic and fibroblastic phenotype. However, with the advent of IHC staining and electron microscopy, as well as cytogenetic and molecular diagnostic studies, MFH is no longer the correct term [2,3] because the characterization of MFH and its subtypes was based completely on H&E staining [8]. As the World Health Organization introduced the term UPS to replace MFH in 2002 [9], the term UPS is used in the case reported here.

UPS in animals has been reported previously in various species including dogs, cats, pigs, cattle, and horses. The lesions involved were of the spleen, head, skin, subcutaneous tissues, ileum, and digits [2,3,6,10,11,12,13,14,15,16,17,18]. Extradural cases are rare in animals despite their commonness in human literature [19,20]. In veterinary literature, extradural undifferentiated sarcoma causing spinal cord compression has only been reported in horses [21]. To the best of the authors’ knowledge, this is the first case report describing extradural UPS in dogs. In the case reported here, clinical signs such as neurologic deficits or ataxia were resulted from the extradural mass causing spinal cord compression or secondary inflammatory responses. This is outlined by improvement of the neurological deficit or gait after surgery. However, vertebral column involvement resulted in rapidly progressing paraparesis leading to paraplegia despite the surgical removal of the mass and surrounding tissues via dorsal laminectomy. Considering the histologic findings, mainly the cytonuclear pleomorphism and mitotic figures, it was assumed to be highly malignant. 

In human medicine, UPSs are classified into five subtypes: undifferentiated high-grade pleomorphic sarcoma, myxofibrosarcoma, undifferentiated pleomorphic sarcoma with giant cells, undifferentiated pleomorphic sarcoma with prominent inflammation, and angiomatoid fibrous histiocytoma. These have been characterized by their specific cytologic and histologic characteristics. In H&E staining, the storiform pattern of pleomorphic spindle cells may indicate UPS [17]. However, as a diagnosis of UPS based on H&E staining is insufficient, the additional diagnostic process of IHC staining was performed to make a definitive diagnosis. Immunological detection for vimentin has been widely used to confirm the mesenchymal origin of soft tissue tumors in both humans and dogs [22]. UPS can be considered as a differential diagnosis if there is vimentin immunoreactivity alone, without any other specific expression of cell line markers [3,17]. In the case reported here, considering H&E and IHC staining observations, a final diagnosis of undifferentiated high-grade pleomorphic sarcoma was made.

It is known that the histologic subtypes of UPS are associated with prognosis in human UPS [2]. In human literature, the prognosis of an undifferentiated high-grade pleomorphic sarcoma is worse than that of a UPS with prominent inflammation but better than that of a UPS with giant cells UPS [2,18]. In human medicine, regional lymph node metastases from UPSs are rare, but the most common site for such metastases is the lung. However, in veterinary medicine, there is less information about the relationship between the histopathological subtypes of UPS and prognosis [18]. UPSs in dogs are considered as locally invasive but not highly metastatic tumors [10,14]. In this case, metastasis to the lung was suspected based on the CT findings at the time of the initial presentation, and metastasis to systemic organs including the heart, lung, spleen, kidney, liver, hilar lymph nodes, skin, and pericystic fat was identified on postmortem microscopic examination. The prognosis is generally considered to be poor once metastatic lesions are identified.

In the current case, total excision was impossible due to the location of the tumor, and the owner refused further adjuvant therapy. Although there are few similar cases, it would seem that an aggressive surgical approach to these tumors, such as vertebrectomy with chemotherapy, should be considered whenever possible. In human medicine, radiation therapy with or without decompressive surgical therapy showed improvements in pain, motor function and urinary continence [23].

In conclusion, in the case reported here, an extradural UPS was invading the vertebral canal resulting in spinal cord damage. Due to the location of the tumor, complete removal was impossible despite a dorsal laminectomy. The dog was euthanized three months postoperatively, and systemic metastasis was confirmed postmortem. To the best of the authors’ knowledge, this is the first case reported of multiple UPS involving extradural mass extending into the spinal cord through a vertebral bone, resulting in damage to the spinal cord, in a dog.

## Figures and Tables

**Table 1:** Postural reaction at the time of presentation (2 = normal; 1 = decreased; 0 = absent).

Postural Reaction			
		Left	Right
Paw position	Front	2	2
	Rear	1	1
Hopping	Front	2	2
	Rear	1	1
Hemistanding and Hemiwalking	Front	2	2
	Rear	1	1
Wheelbarrowing		2	2
Extensor postural thrust		1	1

**Table 2:** Spinal reflex of at the time of presentation. (2 = normal; 1 = decreased; 0 = absent).

Spinal Reflex			
		Left	Right
Patella (femoral)		2	2
Cranial tibial		1	1
Withdrawal	Front	2	2
	Rear	1	2
Perineal	2		

## Data Availability

Not applicable.
